# The evolving gene regulatory landscape—a tinkerer of complex creatures

**DOI:** 10.1186/s13059-021-02412-0

**Published:** 2021-07-08

**Authors:** Geoffrey J. Faulkner

**Affiliations:** 1 Queensland Brain Institute, University of Queensland, Brisbane, QLD 4072 Australia; 2 Mater Research Institute - University of Queensland, TRI Building, Woolloongabba, QLD 4102 Australia

**Editorial**

Our genome is littered with DNA of no clear function. Yet, as shown recurrently by this Special Issue on Regulatory Elements, sequences outside of protein-coding exons are responsible for many, if not most, of the phenotypic differences observed amongst species and individuals. Over the course of evolution, these non-coding regions can provide the raw materials to Jacob’s tinkerer [1], yielding new regulatory switches that shift the spatiotemporal specificity of gene expression. As reviewed by Panigrahi and O’Malley [2], exquisite resolution of genome organisation, accessibility, transcription factor binding, and DNA modifications, now allows the discovery and characterisation of enhancers, promoters, silencers, and other regulatory elements *en masse*. Tracing the evolutionary origins of these elements in different species provides a window into their often similar histories, as well as their present functions. By consequence, this Special Issue of *Genome Biology* is of exceptionally broad biological scope. It presents molecular and computational analyses of diverse experimental systems. The works contained within are however unified by their intent to simplify and better understand the complex relationships between regulatory elements and protein-coding genes.

Transposable elements (TEs) occupy a large proportion of most eukaryotic genomes and, as first shown by McClintock [3], are a major source of gene regulatory innovation. Judd et al. [4] report that the circadian transcriptional network in mouse liver incorporates a number of circadian-responsive TE enhancers contributed by members of the immobile murine RSINE1 family. Focusing on another class of TEs, Troskie et al. [5] use PacBio long-read sequencing to survey the transcriptome of human pseudogenes and identify alternative promoters for various protein-coding genes, such as *RB1*, that are embedded in upstream pseudogenes. A theme of these TE studies, and one shared by the analysis of duplicated gene (ohnolog) regulation in several fish species by Gillard et al. [6], is that TEs spread tissue-specific regulatory elements encoding novel transcription factor binding sites.

Another common thread of this Special Issue is the measurement of protein-coding gene transcription as a readout of regulatory element activity. For instance, the spatial organisation of chromatin is thought to influence the pairing of regulatory elements and protein-coding genes. This is elegantly shown by Furlan-Magaril et al. [7], again using the mouse liver circadian cycle, but here surveying chromosome conformation at high resolution to reveal the influence of differential promoter-enhancer interactions upon gene expression within topologically associated domains. Intriguingly, their results, as well as those of Huang et al. [8] obtained by studying androgen receptor-dependent enhancers in prostate cancer, suggest transcription factors can govern enhancer interactions with promoters. Transcription factors can also attract histone modifying complexes to enhancers. As an example of how perturbed histone modifications can lead to widespread aberrant enhancer activity, Di Giorgio et al. [9] find downregulation of the histone deacetylase *HDAC4* in senescent cells leads to H3K27 hyperacetylation of enhancers linked to senescence-associated genes. Shifting focus to more direct regulatory pathways, Spiegel et al. [10] and Schwich et al. [11] explore the impact of proteins binding to G-quadruplex structures and alternative polyadenylation sites at gene 5′ and 3′ termini, respectively. Finally, Lee et al. [12] survey the regulatory element mutational landscape for thousands of whole cancer genomes and find significant associations between specific cancer subtypes and mutations in certain regulatory elements.

In the near future, more articles will be added to this Special Issue. Together, these works foster a more complete understanding of regulatory element function and evolution, as well as their impact on physiology and disease.
